# A fruit fly model for studying paclitaxel-induced peripheral neuropathy and hyperalgesia

**DOI:** 10.12688/f1000research.13581.2

**Published:** 2018-10-16

**Authors:** Zina Hamoudi, Thang Manh Khuong, Tiffany Cole, G. Gregory Neely

**Affiliations:** 1 University of Sydney, Charles Perkins Centre and School of Life and Environmental Sciences, Camperdown, New South Wales, Australia; 2 University of Sydney, Dr. John and Anne Chong Lab for Functional Genomics, Camperdown, New South Wales, Australia

**Keywords:** Drosophila, fruit fly, paclitaxel, nociception, pain, CIPN

## Abstract

**Background**: Paclitaxel-induced peripheral neuropathy is a common and limiting side effect of an approved and effective chemotherapeutic agent. The cause of this nociception is still unknown.

**Methods**: To uncover the mechanism involved in paclitaxel-induced pain, we developed a *Drosophila* thermal nociceptive model to show the effects of paclitaxel exposure on third instar larvae.

**Results**: We found that paclitaxel increases heat nociception in a dose-dependent manner, and at the highest doses also obstructs dendritic repulsion cues.

**Conclusions**: Our simple system can be applied to identify regulators of chemotherapy-induced pain and may help to eliminate pain-related side-effects of chemotherapy.

## Introduction

Chemotherapy-induced peripheral neuropathy (CIPN) is a dose-limiting side effect of many effective cancer treatments ( Burton *et al.*, 2007), and can have a lasting impact on the quality of life of cancer survivors ( Hausheer *et al.*, 2006 and Shimozuma *et al.*, 2012). A meta-analysis of 31 studies from over 4000 chemotherapy-treated patients revealed that CIPN was prevalent in 68.1% of patients in the first month following chemotherapy, in 60% of patients at 3 months, and in 30% at 6 months or more ( Seretny *et al.*, 2014).

Paclitaxel has a potent ability to cause CIPN ( Addington & Freimer, 2016; Reyes-Gibby *et al.*, 2009). Derived from the bark of the western yew, *Taxus brevifolia*, it is an approved and effective treatment against breast, ovarian, lung and Kaposi sarcoma ( Chang *et al.*, 1993; Gill *et al.*, 1999; Holmes *et al.*, 1991; McGuire *et al.*, 1989; Wani *et al.*, 1971). Patients treated with paclitaxel experience side effects as early as one to three days following treatment ( Lipton *et al.*, 1989; Reyes-Gibby *et al.*, 2009). Common symptoms are hyperalgesia, hypoalgesia, allodynia, tingling, numbness, and shooting pain ( Boland *et al.*, 2010). Paclitaxel has a direct effect on Schwann cells, promotes axonal degeneration, and can cause mitochondrial damage ( André *et al.*, 2000; Cavaletti *et al.*, 1995; Sahenk *et al.*, 1994), however the molecular mechanisms causing pain are still largely unknown.

While much knowledge has been gained about the genetics of pain from vertebrate systems, high-throughput dissection of pain is possible using the fruit fly *Drosophila melanogaster* ( Neely *et al.*, 2010). When challenged with a noxious thermal stimulus, third instar larvae exhibit an aversive escape response that has been utilised to identify conserved genes required for nociception ( Babcock *et al.*, 2009; Neely *et al.*, 2010; Tracey *et al.*, 2003). This nociceptive response is a result of activating class IV multidendritic-dendritic arborisation (md-da) sensory neurons at the site of stimulation ( Hwang *et al.*, 2007). Previously in *Drosophila*, paclitaxel has been reported to be toxic in somatic cells, and causes loss of axons in peripheral nerves. ( Bhattacharya *et al.*, 2012; Cunha *et al.*, 2001). However, its effects on nociception have not yet been evaluated. Here, we examined the effects of paclitaxel exposure on the fruit fly larval nociception system, and observed a robust and dose-dependent increase in pain perception. This system is amenable to high throughput screening and genetic manipulation ( Honjo, *et al*., 2016), and may help define why chemotherapies such as paclitaxel cause pain.

## Methods

### *Drosophila* treatment

All flies were reared at 25°C and 65% humidity over a 12-hour light-dark cycle. Six female and two male *Canton S Drosophila melanogaster* were mated on food medium (5.4% sucrose, 3.6% yeast, 1% agar, 1.2% nipagin, and 0.6% propionic acid) treated with ethanol (vehicle), 0 µM, 0.1 µM, 0.5 µM, 2.5 µM, 5 µM or 10 µM paclitaxel (Taxol®; Catalog No. A4393) purchased from ApexBio (Houston, USA). A stock of 1000 µM paclitaxel in ethanol was prepared and diluted in food medium accordingly to create the different drug concentrated food. F0 Flies were discarded two days after mating and F1 larvae were left to grow for another three days. On the sixth day, early third instar were collected to assess nociception or dendritic morphology.

### Behavioural assay

For the thermal nociceptive assay ( Tracey *et al.*, 2003), distilled water was added to experimental vials to soften the food and release the foraging third instar larvae. The softened, liquid food was then passed through mesh to catch the larvae to be transferred to a 100mm petri dish sprayed with distilled water. The larvae were touched laterally on abdominal segments four to six with a heat probe (soldering iron with narrow tip) set to 42°C or 46°C. The rolling response was measured in seconds with a cut-off of 10 seconds. For each drug concentration, five repeats were performed, with 30–40 larvae per repeat.

### Live confocal microscopy and image analysis

Third instar larvae ( *ppk-Gal4,20xUAS-mCD8-GFP*) were collected, washed, and placed dorsal side up on a microscope slide, immobilized in 1:5 (v/v) diethyl ether to halocarbon oil and covered with a 22 × 50 mm glass coverslip ( Das *et al*., 2017). A Nikon C2 Confocal microscope was used to image GFP-expressing class IV md-da sensory neurons at abdominal segment 2 (A2), under a 20x magnification. Images of Z-stack sections were captured at 1024 × 1024 pixel resolution and representative images were captured at 2048 × 2048 pixel resolution, both with 2x averaging. Z-stack images were converted to maximum intensity projection using ImageJ and automated Sholl analysis was performed on these images. Terminal branches were counted manually. 13 animals were imaged for each treatment. All experiments were conducted in a blinded manner.

### Statistical analysis

Data represent mean ± SEM and are compared to vehicle control. Analysis was done using GraphPad Prism 5. Statistical analysis for response time was done using Krustal-Wallis, followed by Dunn’s pairwise test for multiple comparisons. Statistical analysis for area under the curve mean, terminal branches, critical radius and maximum branches was done using Student’s *t*-test. n.s. p > 0.05. *p < 0.05. **p < 0.01. ***p<0.001.

## Results

**Figure 1.**
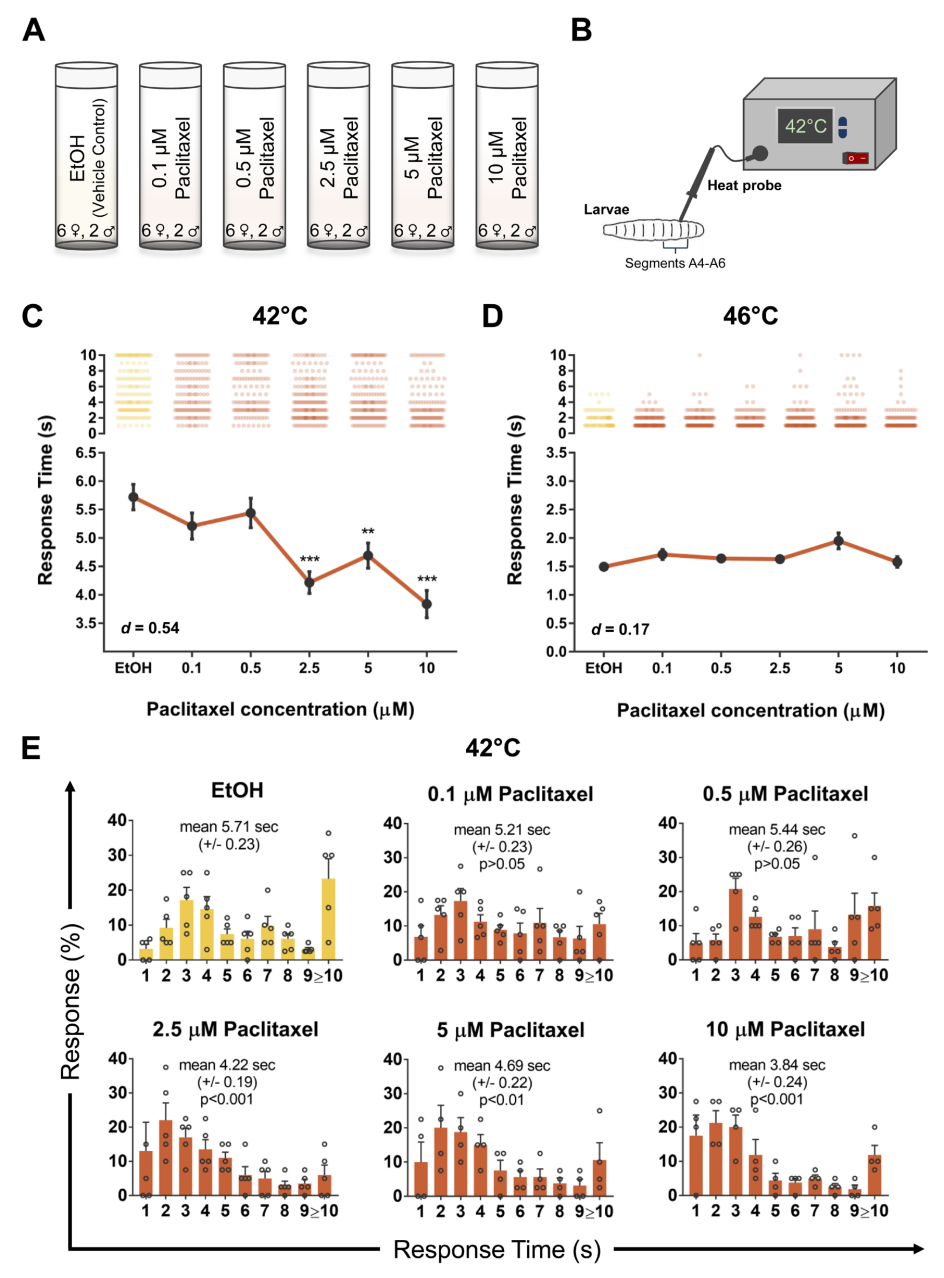
Paclitaxel induces heat-hyperalgesia in *Drosophila* larvae. Schematic representation of the **A**) experimental design and **B**) thermal nociceptive assay in *Drosophila* larvae. **C**– **D**) Average nociceptive latency (in seconds) in response to a 42°C or 46°C thermal stimulus, respectively. Increased paclitaxel concentration significantly induces heat-hyperalgesia in third instar larvae at 42°C. Note concentrations higher than 10 µM paclitaxel were 100% lethal. **E**) Percentage response to each time point in seconds to 42°C thermal stimulus. All values represent mean ± SEM. p values were generated using Krustal-Wallis, followed by Dunn’s pairwise test for multiple comparisons. Significance is relative to vehicle control. Five repeats were performed for each drug concentration with roughly 30 larvae each (n = 130–180 animals).

Our goal here was to develop a reproducible paradigm to investigate the effects of paclitaxel on nociception in the fly larvae. Based on previous studies for toxicity ( Bhattacharya *et al.*, 2012; Cunha *et al.*, 2001), we selected paclitaxel doses below the lethal limit ( Figure 1A), and then tested larval nociception using a heat probe set to a low intensity noxious heat (42°C; Figure 1B), which is mildly nociceptive to fly larvae ( Babcock *et al.*, 2009). Our dose-response study revealed 2.5 µM paclitaxel was sufficient to induce significant hyperalgesia, with a maximal hyperalgesia effect observed at 10 µM ( Figure 1C, *d* = 0.54). Concentrations higher than 10 µM paclitaxel were 100% lethal (not shown). Paclitaxel did not significantly alter heat nociception latency to a 46°C heat stimulus across any of the doses ( Figure 1D, *d* = 0.17). Vehicle (ethanol) control and normal (no ethanol) control showed a response time of 5.71 sec (±0.23 SEM; n=173) and 5.62 sec (±0.20 SEM, n=180, not shown), respectively (42°C; Figure 1E). At low concertation’s of 0.1 µM (5.21 sec ± 0.23 SEM; n=150) and 0.5 µM (5.44 sec ± 0.26 SEM; n=131) paclitaxel did not affect response profiles, however, concentrations of 2.5 µM paclitaxel (4.22 sec ± 0.19 SEM; n=180; p<0.001) and higher altered response distribution and significantly enhanced nociceptive latency (42°C; Figure 1E). The fastest latency response was observed at 10 µM paclitaxel (3.84 sec ± 0.24 SEM; n=140; p<0.001) with a 36.6% increase in response time relative to vehicle control ( Figure 1C).

**Figure 2.**
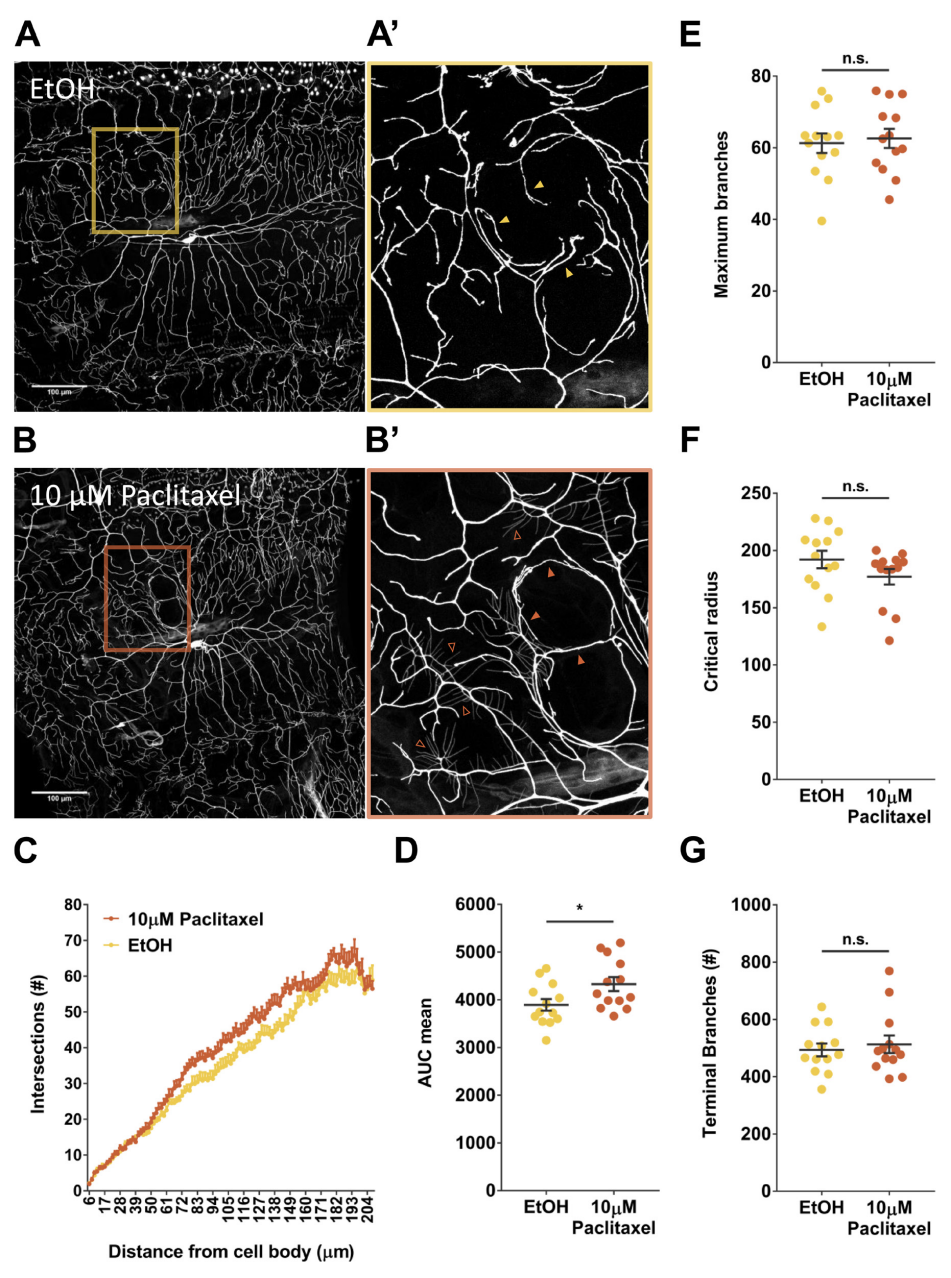
Paclitaxel obstructs dendritic repulsion cues. Representative images ( **A**– **B**) and quantification ( **C**– **G**) of *ppk-Gal4,20xUASmCD8-GF* *P* larvae following vehicle control or 10 µM paclitaxel treatment. Images are of class IV md-da neurons at abdominal segment A2, under a 20x magnification. Scale bar represents 100 µm. Paclitaxel treatment obstructs dendritic repulsion cues (B’, shaded arrowhead), compared to vehicle control (A’). **C**) Branch distribution using Sholl analysis. **D**) Area under the curve. **E–F**) Maximum branch numbers and critical radius reported by Sholl analysis. **G**) Branch terminal numbers. Values represent mean ± SEM (n = 13 animals). n.s. p > 0.05, t tests and post hoc comparisons: *p < 0.05.

To evaluate if paclitaxel exposure caused robust morphological differences in peripheral pain sensing neurons, we fed genetically labelled ( *ppk-Gal4,20xUAS-mCD8-GFP*) larvae paclitaxel and imaged the sensory neuron structure ( Figures 2A–B). Treating larvae with 10 µM paclitaxel affected its repulsive cues with like neurons, overlapping and forming a closed circular structure ( Figure 2B, orange box) compared to vehicle control (Observed in 5 paclitaxel treated animals compared to 0 control animals, Fisher’s Exact Test p < 0.05). In some paclitaxel treated larvae we observed very short dendritic arbors with lower GFP intensity ( Figure 2B’, open arrowhead). This was not observed in vehicle control larvae ( Figure 2A’). We next used Sholl analysis to quantify branch distribution with a focus on number of intersections as a function of distance from the cell soma. This revealed increased branching closer to the cell soma in paclitaxel treated larvae compared to control ( Figure 2C). Area under the curve (AUC) was also calculated for each animal and mean AUC was also plotted for vehicle control (3894 ± 122, n=13) and 10 µM paclitaxel treatment (4329 ± 145.7, n=13) ( Figure 2D). Treatment with paclitaxel significantly increased the area under the curve compared to vehicle control ( Figure 2D, p < 0.05). We also determined maximum branch number and its critical radius and found paclitaxel treatment compared to vehicle control did not have a significant effect on maximum branch number (62.62 ± 2.69; n=13 control and 61.28 ± 2.72; n=13 paclitaxel) or critical radius (177.1 ± 6.78; n=13 control and 192.1 ±7.70; n=13 paclitaxel) ( Figures 2E–F). Finally, paclitaxel did not significantly affect terminal branch number compared with vehicle control ( Figure 2G).

Larval response time in seconds to 42°C heat stimulus Paclitaxel fed larvae were touched with a 42°C heat probe and their response time was measured in seconds with a cut-off of 10 seconds. Different treatments were tested: food control, ethanol control, 0.1 µM, 0.5 µM, 2.5 µM, 5 µM, and 10 µM paclitaxel. Five repeats were performed (n = 130 - 180). Click here for additional data file. Copyright: © 2018 Hamoudi Z et al. 2018 Data associated with the article are available under the terms of the Creative Commons Zero "No rights reserved" data waiver (CC0 1.0 Public domain dedication).

Larval response time in seconds to 46°C heat stimulus Paclitaxel fed larvae were touched with a 46°C heat probe and their response time was measured in seconds with a cut-off of 10 seconds. Different treatments were tested: food control, ethanol control, 0.1 µM, 0.5 µM, 2.5 µM, 5 µM, and 10 µM paclitaxel. Five repeats were performed (n = 130 - 180). Click here for additional data file. Copyright: © 2018 Hamoudi Z et al. 2018 Data associated with the article are available under the terms of the Creative Commons Zero "No rights reserved" data waiver (CC0 1.0 Public domain dedication).

Dendritic morphology of third instar *ppk-Gal4,20xUASmCD8-GFP* Confocal images of vehicle control and 10 µM paclitaxel treated larvae. Images represent class IV md-da neurons at abdominal segment A2. Images are at 20x magnification with 2x averaging. Scale bar represents 100 µm. Click here for additional data file. Copyright: © 2018 Hamoudi Z et al. 2018 Data associated with the article are available under the terms of the Creative Commons Zero "No rights reserved" data waiver (CC0 1.0 Public domain dedication).

## Discussion

Here we report a simple, high-throughput genetically tractable system to dissect the mechanisms of CIPN in *Drosophila*. Some effective and common chemotherapeutic agents such as paclitaxel cause peripheral neuropathy in a dose-dependent manner, limiting its therapeutic potential. Hyperalgesia, hypoalgesia and allodynia are some of the common side effects experienced by patients ( Boland *et al*., 2010). By utilising a conserved hyperalgesia response, we performed a dose-finding study to determine the best drug dose to further investigate mechanisms for how paclitaxel causes pain. Our findings in *Drosophila* larvae are reminiscent of human patients, where paclitaxel increased pain sensitivity in a dose-dependent manner ( Burton *et al*., 2007).

*Drosophila* experience a nociceptive response by activation of class IV md-da neurons at the site of stimulation. These neurons form extensive, space filling dendritic arbors that exhibit repulsive characteristics where they do not overlap with neighbouring dendrites but instead terminate projection or make abrupt turns ( Grueber *et al*., 2007). In our system, we found that treatment with paclitaxel obstructs these dendritic guidance cues, leading to an overlap of dendritic arbors. This may be due to paclitaxel’s effect on mitotic spindles where it binds to beta-tubulin, stabilizing its polymerization, leading to a disruption of the microtubule organization, and thus impacting microtubule-based dendritic guidance ( De Brabander *et al.,* 1981; Parness & Horwitz, 1981; Rowinsky *et al*., 1988; Schiff & Horowitz, 1980). Paclitaxel’s unknown neuropathic mechanism may be related to its effects on microtubule function and axonal transport. Our simple system may be used with genomic approaches to dissect this mechanism and identify regulators of chemotherapy pain. Together this work can lead to a better understanding of how the pain arises, and potentially avoid these severe side effects while more effectively targeting the underlying disease.

## Data availability

The data referenced by this article are under copyright with the following copyright statement: Copyright: © 2018 Hamoudi Z et al.

Data associated with the article are available under the terms of the Creative Commons Zero "No rights reserved" data waiver (CC0 1.0 Public domain dedication). 

**Dataset 1: Larval response time in seconds to 42°C heat stimulus.** Paclitaxel fed larvae were touched with a 42°C heat probe and their response time was measured in seconds with a cut-off of 10 seconds. Different treatments were tested: food control, ethanol (vehicle) control, 0.1 µM, 0.5 µM, 2.5 µM, 5 µM, and 10 µM paclitaxel. Five repeats were performed (n = 130 - 180). DOI, 10.5256/f1000research.13581.d191022 ( Hamoudi *et al.*, 2018a).

**Dataset 2: Larval response time in seconds to 46°C heat stimulus.** Paclitaxel fed larvae were touched with a 46°C heat probe and their response time was measured in seconds with a cut-off of 10 seconds. Different treatments were tested: food control, ethanol (vehicle) control, 0.1 µM, 0.5 µM, 2.5 µM, 5 µM, and 10 µM paclitaxel. Five repeats were performed (n = 130 - 180). DOI, 10.5256/f1000research.13581.d191023 ( Hamoudi *et al.*, 2018b).

**Dataset 3: Dendritic morphology of third instar *ppk-Gal4,20xUASmCD8-GFP*.** Confocal images of vehicle control and 10 µM paclitaxel treated larvae. Images represent class IV md-da neurons at abdominal segment A2. Images are at 20x magnification with 2x averaging. Scale bar represents 100 µm. DOI, 10.5256/f1000research.13581.d222127 ( Hamoudi *et al.*, 2018c).
