# Supplementary figures and images for: A fruit fly model for studying paclitaxel-induced peripheral neuropathy and hyperalgesia
Source: F1000Res. 2018 Oct 16;7:99. Originally published 2018 Jan 23. [Version 2] doi: 10.12688/f1000research.13581.2 (PMC6402077; doi:10.12688/f1000research.13581.2)

10  $\mu$ M Paclitaxel

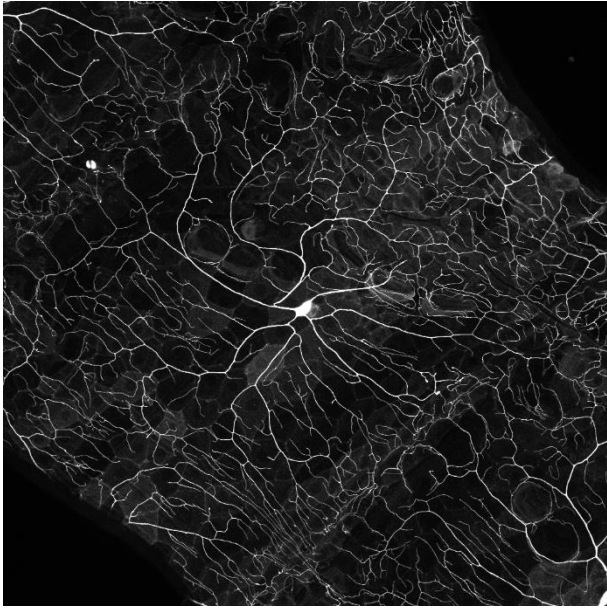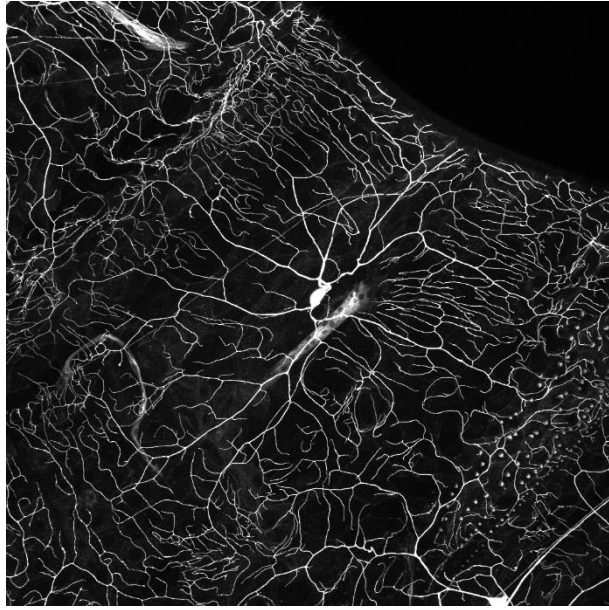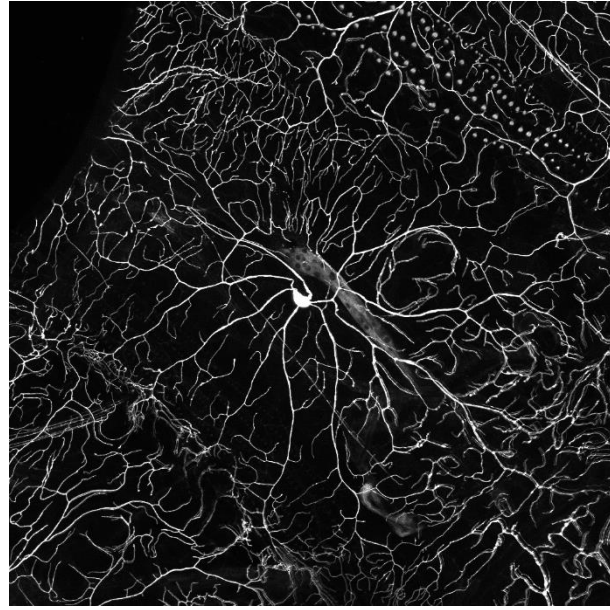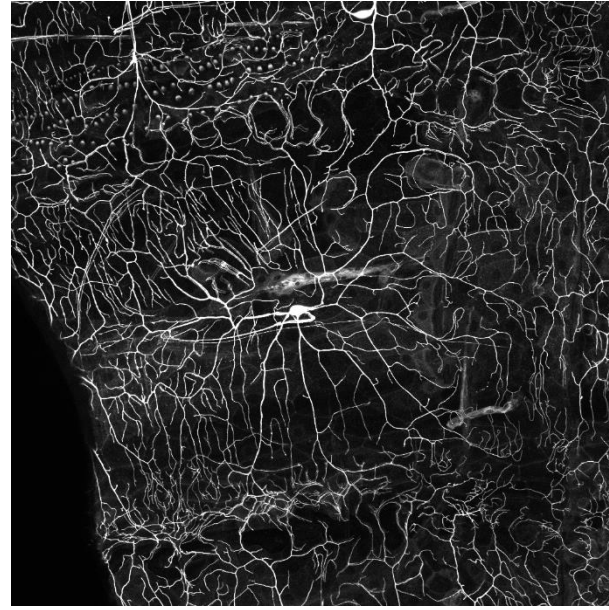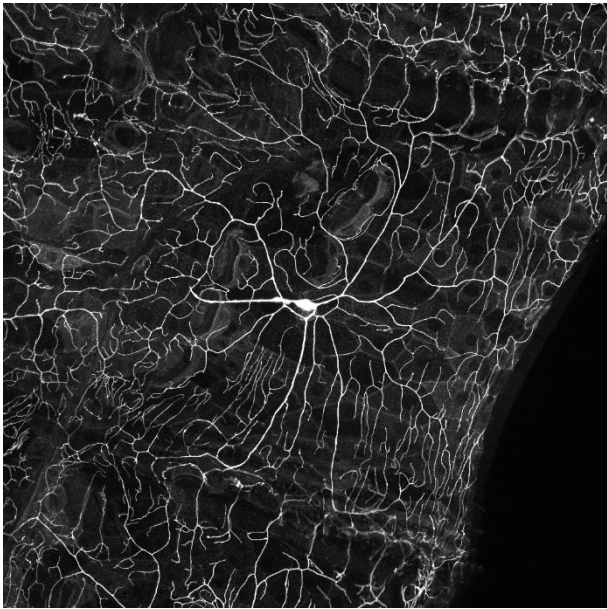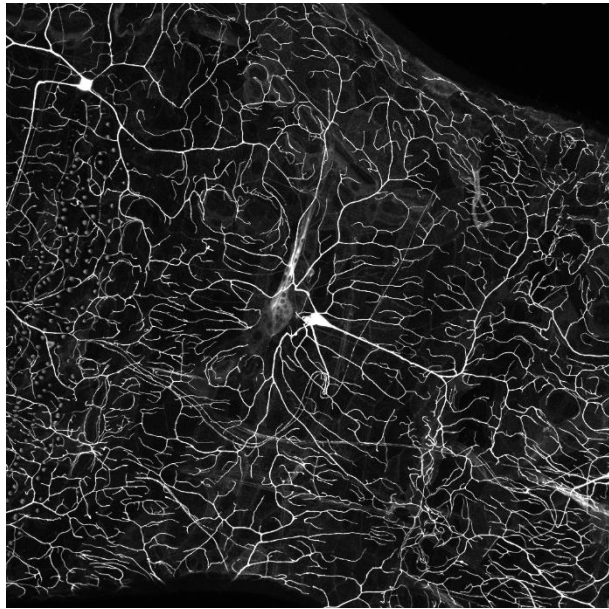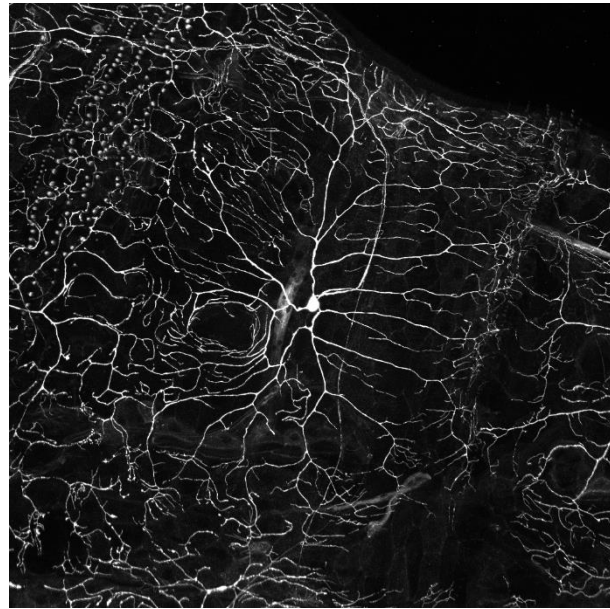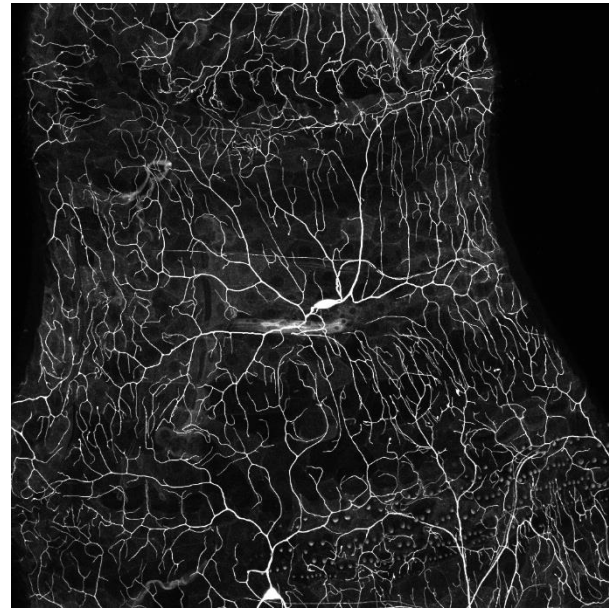

10  $\mu$ M Paclitaxel

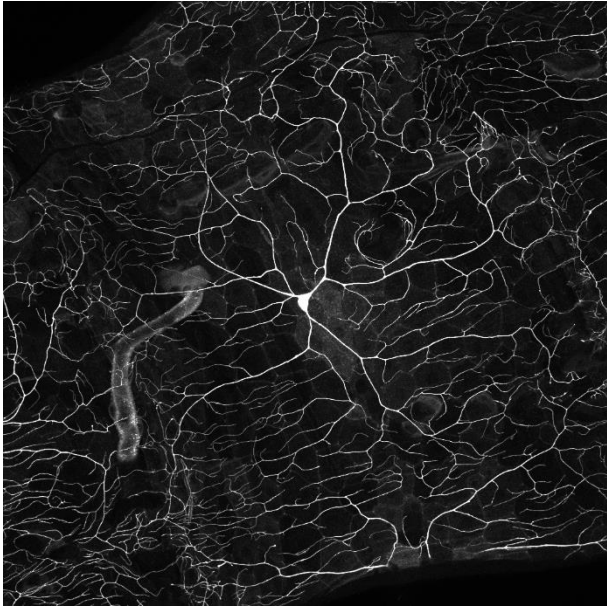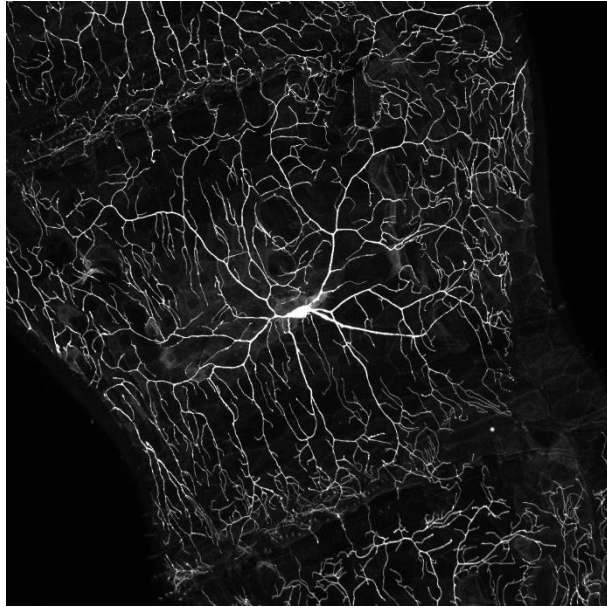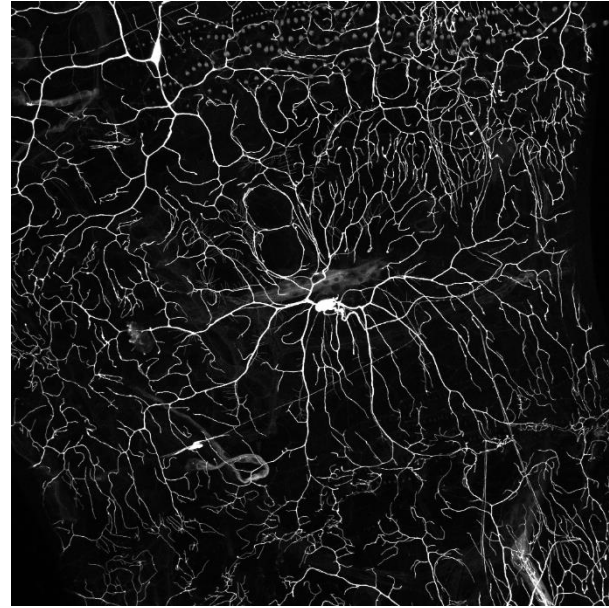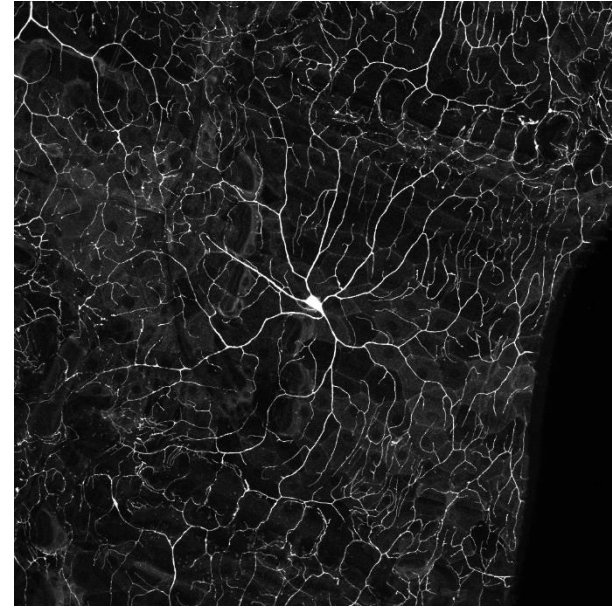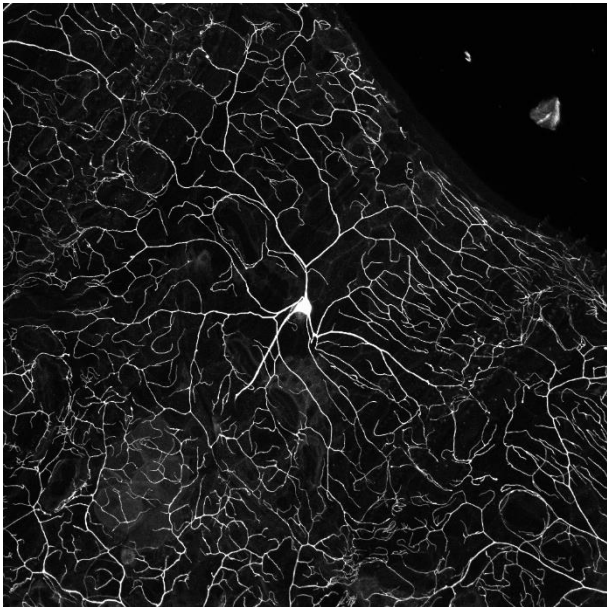

EtOH

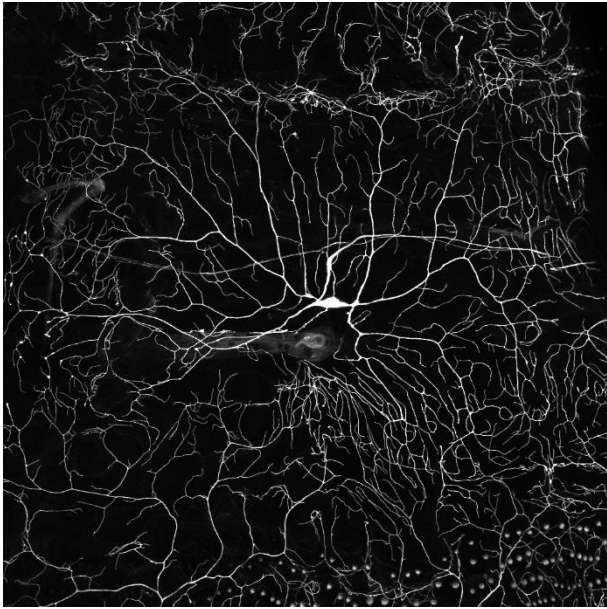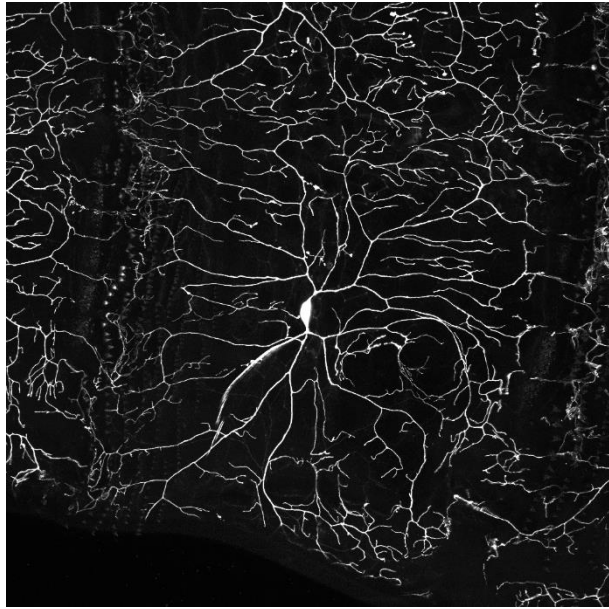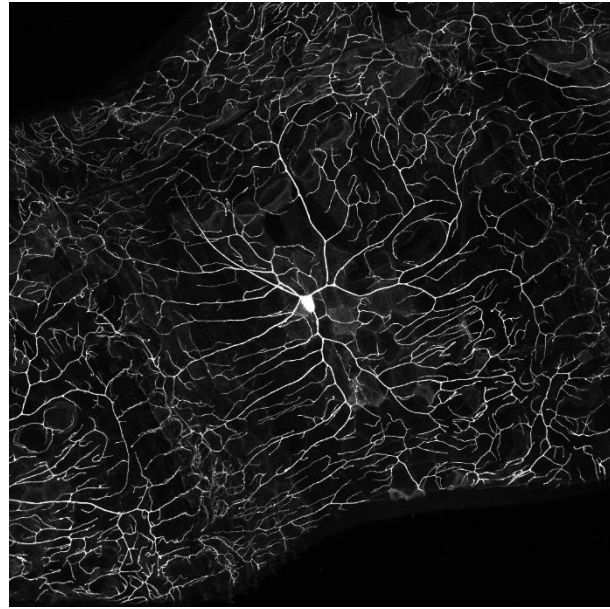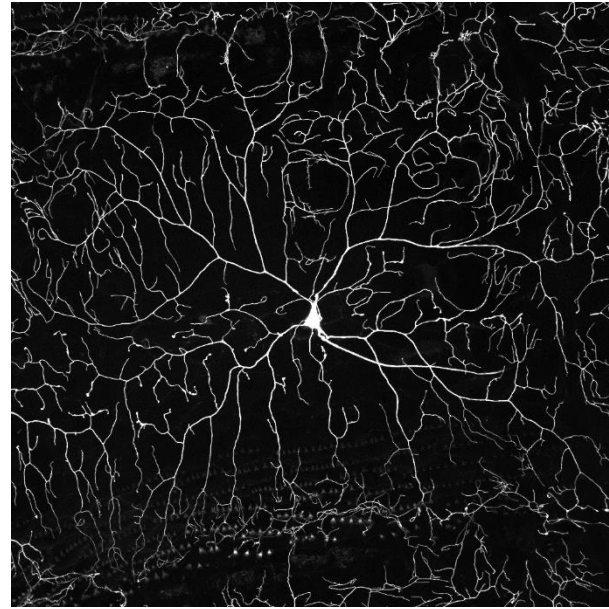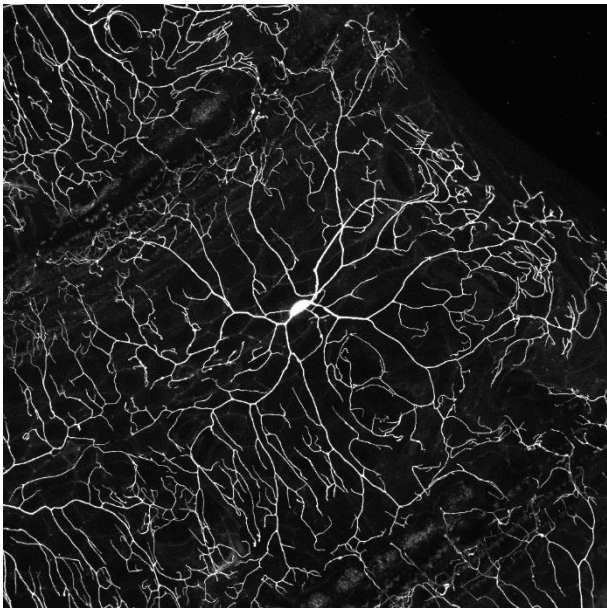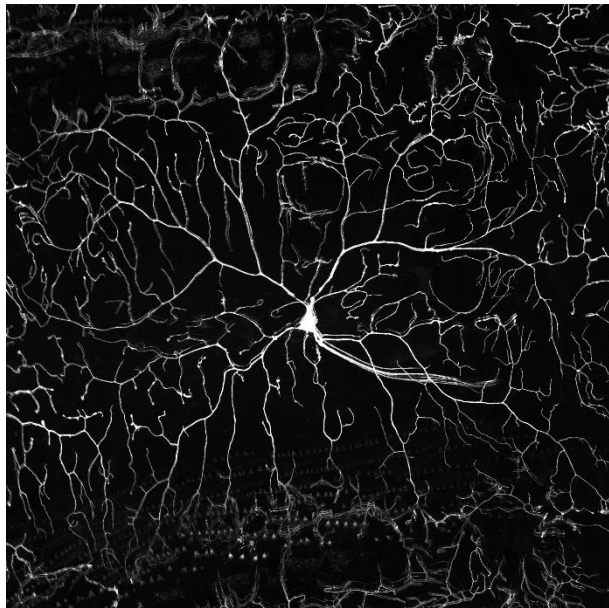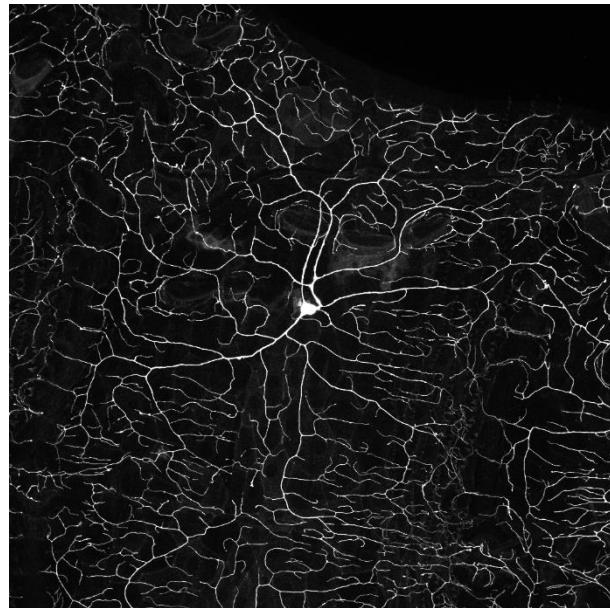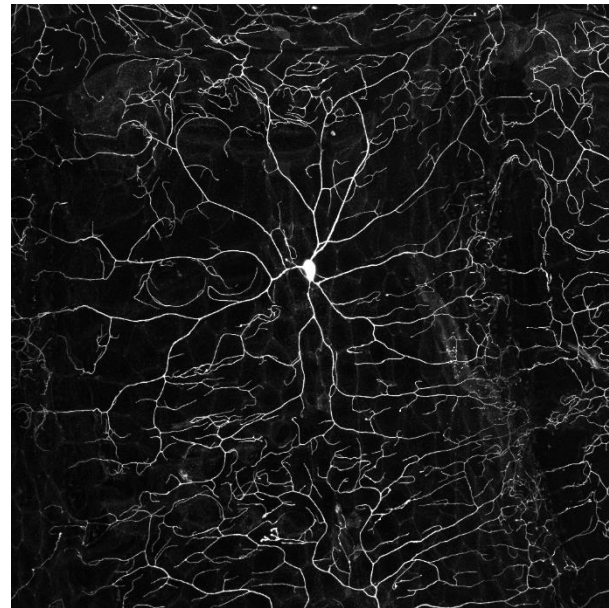

EtOH

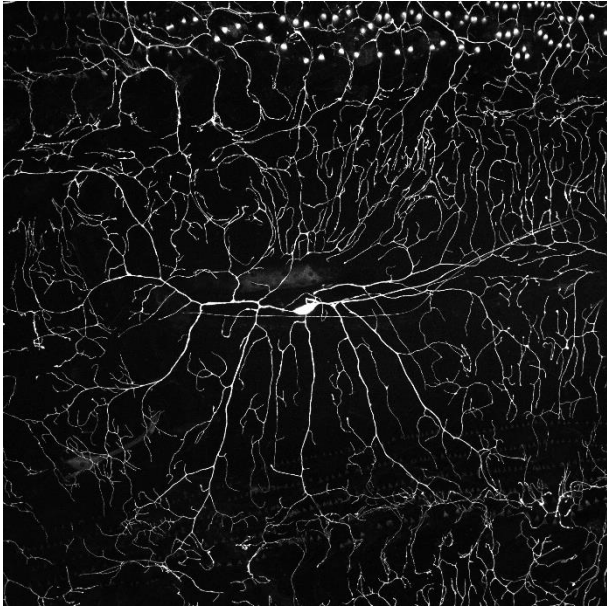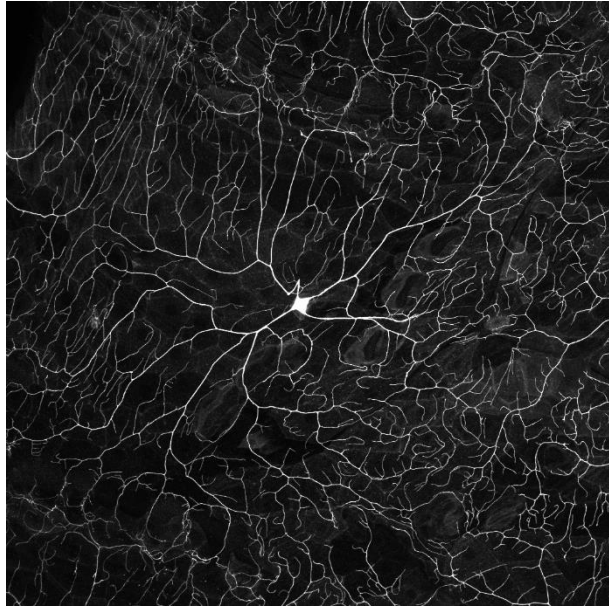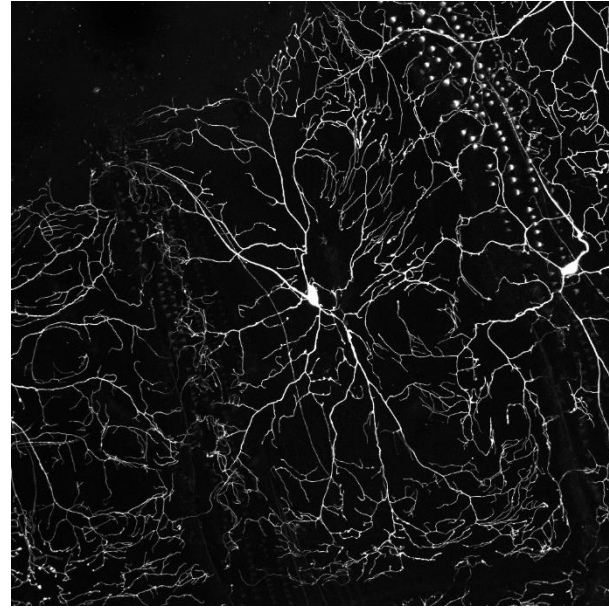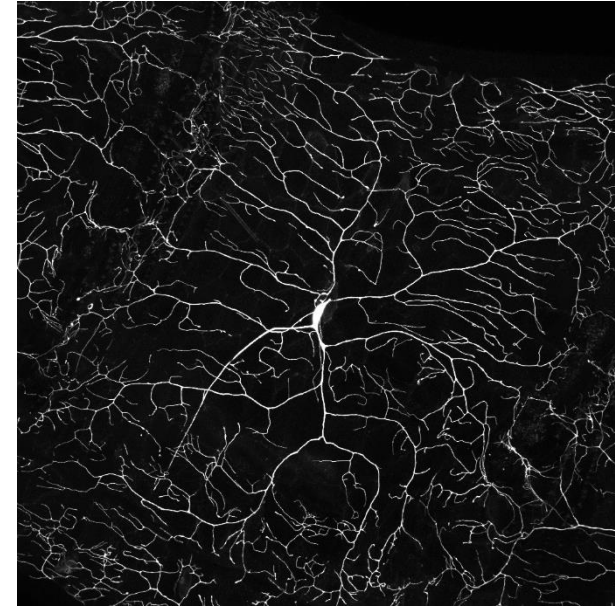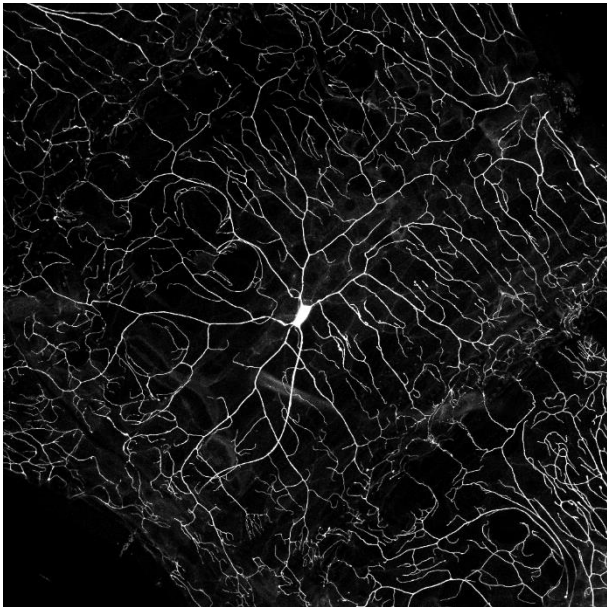

Supplement: Dendritic morphology of third instar ppk-Gal4,20xUASmCD8-GFP — Confocal images of vehicle control and 10 µM paclitaxel treated larvae. Images represent class IV md-da neurons at abdominal segment A2. Images are at 20x magnification with 2x averaging. Scale bar represents 100 µm. [file f1000research-7-18043-s0002.tgz › 3372bf96-1cf0-41c1-9856-b8936e937a9e_dataset_3.pdf]
