# Supplementary material for: A fruit fly model for studying paclitaxel-induced peripheral neuropathy and hyperalgesia
Source: F1000Res. 2018 Oct 16;7:99. Originally published 2018 Jan 23. [Version 2] doi: 10.12688/f1000research.13581.2 (PMC6402077; doi:10.12688/f1000research.13581.2)
Supplement: Peer review history

# Peer review: Reviewer response for version 2

**DOI:** 10.5256/f1000research.18043.r43411

**Published:** 2019-03-04

**Authors:** Giorgio Gilestro

**Affiliations:** 1 Department of Life Sciences, Imperial College London, London, UK

In this work, Hamoudi et al. present the use of Drosophila larvae as a potential biological in-vivo model to study chemotherapy-induced peripheral neuropathy. The work is solid and well presented. The data are convincing and the methods well explained.

 I found only two aspects of the work that are perhaps overlooked and could use a couple of notes in the discussion:

1. The work focuses on the effects of only one drug. The title appropriately refers to paclitaxel indeed, but it would be interesting to speculate on whether we could expect the same response using other drugs too.
2. Larvae are developing organisms. Their neuronal network changes as they grow from instar to instar. CIPN, on the other hand, is normally observed in post-developmental conditions. Can we assume that the changes in synaptic structures reported in figure 2 would be observed in a fully developed nervous system too?

I think adding a couple of lines of speculation regarding point 1 and 2 would strengthen the paper.

I have read this submission. I believe that I have an appropriate level of expertise to confirm that it is of an acceptable scientific standard.

# Peer review: Reviewer response for version 1

**DOI:** 10.5256/f1000research.14752.r30145

**Published:** 2018-03-22

**Authors:** Salahadin Abdi

**Affiliations:** 1 Department of Pain Medicine, Division of Anesthesiology and Critical Care, University of Texas MD Anderson Cancer Center, Houston, TX, USA

This study examined the effects of Paclitaxel exposure on *Drosophila *larval nociception system and the authors propose that their model is suitable for high throughput screening and further mechanistic studies.  The study is overall an interesting and clearly written, however, I do have the following concerns:

 1. The dose response effect of thermal stimulation was only at 42 degrees. There was no discussion or explanation why this effect was not seen at 46 degrees.

 2. The behavior experiment was based on thermal stimulation. I would be interested why mechanical stimulation was not chosen since mechanical sensitivity is common among patients who develop Paclitaxel induced peripheral neuropathy?

 3. It is not clear to me what the timeline is between the exposure of the larvae with paclitaxel and performing microscopic studies.

 4. There should be at least a short discussion about the result.

I have read this submission. I believe that I have an appropriate level of expertise to confirm that it is of an acceptable scientific standard, however I have significant reservations, as outlined above.

# Response

**Published:** 2018-10-09

**Authors:** Greg Neely

**Affiliations:** University of Sydney, Australia

Thank you for your comments.

**Response to comment #1:** This effect is not seen at 46°C. At this temperature intensity, larvae respond rapidly (~1.5 seconds) and it is difficult to see even faster responses. To look for hyperalgesia, we instead lowered the heat stimulus intensity to 42°C, which is at the threshold for nociception in this system, and where nociceptive responses take on average ~5 seconds to elicit.

**Response to comment #2:** The type IV multidendritic nociceptor neurons that transduce heat nociception also transduce mechanical nociception, as these neurons are multimodal. We have tried on numerous occasions to generate reproducible data for mechanical nociception but so far in our hands this assay does not work well enough for us to feel comfortable publishing. Given the multimodal nature of type IV multidendritic nociceptor neurons, we reasoned that thermal hyperalgesia is a good readout for the overall sensitization of these sensory neurons.

**Response to comment #3:** The animals are born into paclitaxel containing food, and then early third instar are collected at day 6 to assess nociception or dendritic morphology. This information was provided in the methods, however we have further clarified this aspect.

**Response to comment #4:** We have now written a short discussion, please see discussion section.

# Peer review: Reviewer response for version 1

**DOI:** 10.5256/f1000research.14752.r30150

**Published:** 2018-03-01

**Authors:** Adam Claridge-Chang

**Affiliations:** 1 Institute of Molecular and Cell Biology (IMCB), Singapore, Singapore

The authors characterize the thermal nociception in *Drosophila* larvae that have been cultured on a range of paclitaxel concentrations. Using a heat probe to elicit the rolling defense behavior, they find that while paclitaxel has no effect on response times with a 46°C probe, it shortens probe response times when the larvae have been grown on 2.5 µm paclitaxel or above.

 The authors and readers might like to consider the following comments on and questions about the 23 Jan 2018 version.

1. The Title describes a model for studying paclitaxel-induced pain, however the assay uses a heat probe to induce pain, and paclitaxel lowers the sensitivity to the probe, thus modeling the hyperalgesia component of paclitaxel CIPN. Would the Title better serve the reader if edited to focus on this side-effect specifically?
2. In **Abstract–Results**, the authors write: "We found that paclitaxel increases pain perception in a dose-dependent manner, without overt morphological changes." Changing "perception" to "sensitivity" would eliminate the baggage of the former word.
3. In **Abstract**– **Conclusions**: "Our simple, high throughput model can be combined with genomics approaches to identify regulators of chemotherapy-induced pain to eliminate its adverse side effects." However, they have not established that this is high-throughput by most common definitions of the term, nor do they show anywhere in the paper that it can be combined with genomics. The Conclusions would be improved if rephrased to better reflect what the data show.
4. "High-throughput" is a phrasal adjective that requires a hyphen.
5. In **Introduction** it says "This system is amenable to high throughput screening" however, this is not shown in the present manuscript nor is a reference cited in support of the statement.
6. "Krustal-Wallis ANOVA."  Correct to "Kruskal–Wallis."
7. I encourage the authors to use estimation statistics instead of significance testing. This would involve presenting and discussing the effect sizes. For example, in Figure 1c, it looks like 2.5 µm paclitaxel has the effect of reducing response time by ~1.5 s.
8. It would be nice to calculate standardized effect sizes (e.g. Cohen's d) of the paclitaxel effects; this would allow the authors and readers to estimate sample sizes needed for a screen (and thus possible throughput rates).
9. In **Results, **the authors write "Thus we establish that paclitaxel sensitizes larvae to heat pain via enhancing sensory neuron or higher order nociception, and not via inducing overt morphological changes." Is it true that enhancing sensory neuron or higher-order nociception are the only two alternatives? If not, this sentence should be rephrased.
10. The **Conclusions **section reads more like an overview of future plans for the assay. Could it be rewritten to more closely address the paper's findings?

I have read this submission. I believe that I have an appropriate level of expertise to confirm that it is of an acceptable scientific standard.

# Response

**Published:** 2018-10-09

**Authors:** Greg Neely

**Affiliations:** University of Sydney, Australia

Thank you for your comments.

**Response to comment #1:** That’s a reasonable point. Since first submission, we have new data that shows our model also involves peripheral neuropathy, so taken together, we have updated the title to capture this aspect and address the reviewer’s comment. The new title is “ **A fruit fly model for studying paclitaxel-induced peripheral neuropathy and** **hyperalgesia**”. We hope this is acceptable.

**Response to comment #2:** Done.

**Response to comment #3: **We have revised this section and now state “Our simple system can be applied to identify regulators of chemotherapy-induced pain”.

**Response to comment #4:** Done.

**Response to comment #5:** We have now included a reference for this statement.

**Response to comment #6:** Done.

**Response to comment #7:** We thank the reviewers for their comment and we have incorporated the estimation statistics into our analysis and added all the data points. 

**Response to comment #8:** We have calculated the effect size, added it to the graphs (1C and 1D), and we have now also mentioned this in the results section. Moreover, we have changed the data representation to show all the data points.

**Response to comment #9:** Good point, this has been revised as suggested.

**Response to comment #10:** We have now added a discussion section.
